# Correction to “Integrating multimodal management and molecular profiling in a patient with BRAF V600E–positive melanoma and brain metastases”

**DOI:** 10.3322/caac.70093

**Published:** 2026-06-05

Gritsch D, Fadden RM, Mahar MK, et al. Integrating multimodal management and molecular profiling in a patient with BRAF V600E‐positive melanoma and brain metastases. *CA* *Cancer J Clin*. 2026;76:e70079. doi:10.3322/caac.70079

Two author names were misspelled in the original work. “Cahil” should be “Cahill” for Daniel P. Cahill III MD, PhD and “Khandsekar” should be “Khandekar” for Melin J. Khandekar MD, PhD.

Emily Sullivan (Massachusetts General Hospital Cancer Center / Harvard Medical School) was inadvertently omitted from the final manuscript. Ms. Sullivan contributed to the radiation necrosis section of the manuscript and all co‐authors are aware of and support this addition. She should be listed between Kevin S Oh, MD MBA and Daniel P Cahill, III, MD PhD, and she has no conflicts of interest to disclose.

A microorganism name misspelling on page 1, paragraph 1, line 9 should be “Clostridioides” instead of “Clostridiodies”.

A terminology error on page 2, paragraph 3, line 3, should be “nonsteroidal anti‐inflammatory drugs” instead of “nonsteroidal noninflammatory drugs”.

We apologize for these errors.

